# Absence of lung sliding is not a reliable indicator of pneumothorax in patients who require high PEEP

**DOI:** 10.1186/cc14219

**Published:** 2015-03-16

**Authors:** J Golub, A Markota, A Stožer, G Prosen, A Bergauer, F Svenšek, A Sinkovič

**Affiliations:** 1 University Medical Centre Maribor, Slovenia; 2 University of Maribor, Slovenia; 3 Community Health Centre, Maribor, Slovenia

## Introduction

The objective of our study was to estimate correlation between PEEP and disappearance of lung sliding (LS) due to lung overdistension in the absence of pneumothorax.

## Methods

We performed a prospective study from September 2013 to May 2014 in adult patients with respiratory failure who required mechanical ventilation, lung CT and recruitment manoeuvre. Lung CT was used as the gold standard to exclude pneumothorax. A staircase recruitment manoeuvre was used with 5 cmH_2_O increases of PEEP from baseline to 35 cmH_2_O and decreases in reverse order. The duration of each step was 1 minute. Lung ultrasound was performed to evaluate LS at each step in one intercostal window in the highest point of left and right hemithoraces by physicians trained in lung ultrasound and blinded to changes in PEEP.

## Results

**Figure 1:**
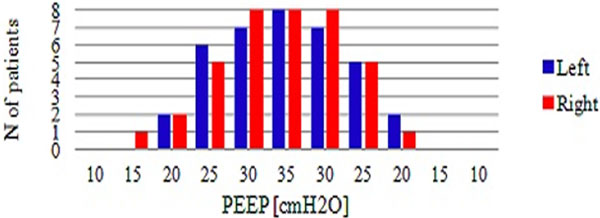
**Absence of LS during increasing and decreasing PEEP**.

**Figure 2:**
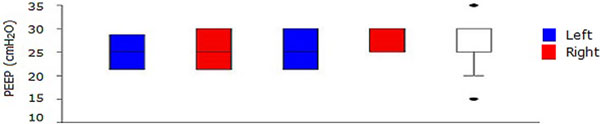
**Values of PEEP above which LS was not present**.

In all, eight patients were included; five (62.5%) males, mean age 70.1 ± 7.4 years. Mean auto-PEEP was 0.7 ± 0.4 cmH_2_O. The values of PEEP at which LS disappeared or reappeared were compared using the Wilcoxon signed-rank test to assess the influences of anatomical side and PEEP increase or decrease. The values of PEEP at disappearance of LS for the right lung were not statistically significantly different from the left lung (*P *= 0.844 for increases, *P *= 0.938 for decreases). The values of PEEP at which LS disappeared obtained during increases were not statistically significantly different from values obtained during decreases (*P *= 1.000 for left lung, *P *= 0.875 for right lung; Figure 1). From data pooled from both sides and protocols, the median value of PEEP at which LS disappeared as a false positive sign of pneumothorax was 25 cmH_2_O (interquartile range = 20 to 30 cmH_2_O). At PEEP = 10 cmH_2_O, no patient showed absence of LS, whereas at PEEP = 35 cmH_2_O, all patients showed absence of LS (Figure 2).

## Conclusion

According to this study, higher PEEP levels correlate with disappearance of LS without pneumothorax. Absence of LS in patients with high PEEP should be interpreted with caution and other signs of pneumothorax should be sought before therapeutic interventions are attempted.

